# Comparison of Calf Muscle and Achilles Tendon Stiffness Between Triathletes and Physically Active Controls: A Cross-Sectional Study Using Shear Wave Elastography

**DOI:** 10.1155/tsm2/2468866

**Published:** 2025-04-09

**Authors:** Estevão de Souza Diniz, José Roberto de Souza Júnior, Pedro Bainy Franz, Leandro Gomes de Jesus Ferreira, Amanda Morais Costa, Julia Rocha, Ana Luísa Ribeiro, Leandro Moreira, Isabella da Silva Almeida, Jeam Marcel Geremia, Fernando Diefenthaeler, Marco Aurelio Vaz, Rodrigo Scattone Silva, João Luiz Quaglioti Durigan, Rita de Cássia Marqueti

**Affiliations:** ^1^Laboratory of Muscle and Tendon Plasticity, Faculty of Health Sciences and Technologies, University of Brasília, Federal, Brazil; ^2^Laboratory of Molecular Analysis, Faculty of Health Sciences and Technologies, University of Brasília, Federal, Brazil; ^3^Physical Therapy Department, University Center of the Federal District, Federal, Brazil; ^4^Research Laboratory of Exercise, School of Physical Education, Physiotherapy, and Dance, Federal University of Rio Grande do Sul, Porto Alegre, Brazil; ^5^Biomechanics Laboratory, Federal University of Santa Catarina, Florianópolis, Brazil; ^6^Brazilian Tendinopathy and Sports Injuries Research Group, Federal University of Rio Grande do Norte, Santa Cruz, Brazil

**Keywords:** Achilles tendon, muscle stiffness, physical fitness, triathlon, ultrasound elastography

## Abstract

**Introduction:** Triathlon's global popularity, with over 120 national federations and millions of athletes, has led to an increase in injuries, particularly Achilles tendinopathy, affecting 12−24% of long-distance and 7.7% of short-distance triathletes. Understanding the morphological adaptations of the Achilles tendon and calf muscles is crucial.

**Objective:** This study compares the stiffness of the Achilles tendon and calf muscles between triathletes and physically active controls, while identifying differences in the superficial, middle, and deep layers of the calf muscles across and within these groups.

**Methods:** A cross-sectional study was conducted with 42 participants divided into four groups: 10 male triathletes, 10 male controls, 11 female triathletes, and 11 female controls. Shear wave elastography assessed the stiffness of the Achilles tendon and calf muscles.

**Results:** No between-group differences were found for the overall stiffness of the Achilles tendon and calf muscles. In the soleus muscle, the stiffness of the superficial layer was greater in the male control group compared to female triathletes (*p*=0.002). Also, the middle layer was greater in the male control group compared to the male triathletes (*p*=0.023) and female triathletes (*p*=0.028). Finally, the middle layer was greater in male controls compared to female triathletes (*p*=0.008). Within-group differences showed that the superficial layer was generally stiffer than the middle and deep layers across all groups and muscles. Notably, in the lateral gastrocnemius, the deep layer showed greater stiffness compared to the middle layer only for female controls (*p*=0.014).

**Conclusion:** Triathlon does not affect the overall stiffness of the Achilles tendon and calf muscles, but differences in muscle layers highlight the need for a segmented approach in elastography, which may reveal specific training effects or injury risks.

## 1. Introduction

Triathlon is a growing sport worldwide, with 120 national federations affiliated with the International Triathlon Union [1]. In 2023, more than 200,000 athletes participated in global qualifying events, with a notable increase in female participation. The number of women in the Ironman World Championship increased from 560 in 2011 to 1800 in 2022, highlighting the sport's growing inclusivity. The average age of participants, 42 years, underscores the sport's accessibility to diverse age groups [2, 3]. Alongside this popularization, there has also been an increase in the number of injuries, especially due to overuse of the lower limbs, with the highest incidences in the ankle (12%–24%), foot (14%), and knee (56%) [4]. Achilles tendinopathy is particularly prevalent among elite athletes, affecting 12%–24% of long-distance triathletes compared to their Olympic distance counterparts [5]. This condition is characterized by pain, local thickening, and reduced functional capacity, resulting from the Achilles tendon's small cross-sectional area and high axial loading forces [6–8].

The stiffness of the Achilles tendon can be altered in the presence of injury [9]. Thus, to prevent Achilles tendinopathy, understanding the structure of the Achilles tendon and calf muscles is crucial. Shear wave elastography (SWE), a method studied and validated by several authors [9–13], can be employed for this purpose. The SWE method has been applied to assess different tendons and muscles of the upper (i.e., rotator cuff; biceps brachii) and lower limbs (i.e., patellar; vastus medialis) [9, 12, 14]. Previous studies have found moderate evidence of this technique to differentiate pathologic from healthy Achilles tendons. Also, high correlations were found with the score of the VISA-A scale in participants with Achilles tendinopathy [11].

Despite the widely use of SWE, no study was conducted to assess the stiffness of the Achilles tendon and calf muscles in triathletes. Endurance sports significantly affect tendon and muscle stiffness, particularly in the Achilles tendon, a critical structure for running economy. Therefore, it is expected that prolonged and intense training can lead to stiffness reduction and elevated injury risks, especially in older athletes [15]. Also, these adaptations can vary by sex, as female athletes often exhibit lower tendon stiffness than males [16, 17]. Despite these known differences, gaps remain regarding triathlon-specific adaptations and tailored protocols for female athletes [18]. Finally, no study assessed the differences on stiffness according to muscle layers in this population. Type I collagen primarily increases stiffness in superficial layers, while Type III collagen enhances elasticity in deeper layers [19]. These structural differences may influence calf muscle stiffness and, consequently, affect the risk of developing Achilles tendinopathy and remain to be determined.

We, therefore, aimed to compare the stiffness of the Achilles tendon and calf muscles between triathletes of both sexes and healthy physically active controls. The secondary aim was to compare the stiffness of the superficial, middle, and deep layers of the calf muscles between these groups. Finally, we aimed to identify differences in the stiffness among the layers of the calf muscles within each group. The hypothesis was that triathletes would present lower stiffness of the Achilles tendon and calf muscles compared to active and healthy controls, due to the training load and overuse of these structures. Additionally, it was expected that triathletes would show lower stiffness of the superficial, middle, and deep layers of the calf muscles compared to controls for the same reason. Finally, it was expected that the superficial layer of the calf muscles would present greater stiffness compared to the other layers within each study group.

## 2. Methods

### 2.1. Study Design

This cross-sectional study followed the recommendations of STROBE (Strengthening the Reporting of Observational Studies in Epidemiology) and was approved by the Ethics Committee on Research with Humans of the University of Brasília (CAAE: 70162723.0.0000.8093). Informed consent was obtained in accordance with the Helsinki Declaration and local resolution.

### 2.2. Participants

Forty-two participants were selected for this study (twenty-one triathletes and twenty-one healthy controls), recruited nonrandomly using a combination of strategies. Partnerships were established with the Brazilian Triathlon Confederation (BrasilTri), regional triathlon federations, and companies organizing triathlon competitions in the region. Recruitment was also promoted via social media advertisements and posters distributed at higher education institutions and gyms in the Federal District.

Interested individuals completed an online questionnaire collecting demographic data, sports history, training volume and intensity, competitive performance, and injury history. The questionnaire also included questions about training monitoring apps, such as Strava and Garmin Connect, and required proof of their best running pace in triathlon competitions within the past 12 months. Eligibility was determined based on predefined criteria, and selected participants were invited for evaluations at the Laboratory of Musculotendinous Plasticity (LaPlast) at the University of Brasília.

Participants were divided into four groups: male triathletes (*n* = 10), male controls (*n* = 10), female triathletes (*n* = 11), and female controls (*n* = 11). The triathlon group included athletes of age greater than 18 years old, of both sexes, and who had practiced the sport for more than a year with a frequency training of at least four times a week. Triathletes were instructed not to train in the 8 h preceding their scheduled evaluation. All evaluations were conducted in the morning to facilitate and standardize the assessment period. A longer training abstention period or control over their training phase was not requested to avoid disrupting the athletes' routine, which could have led to withdrawal from the study.

The control group included healthy, physically active participants with age greater than 18 years old, of both sexes, and who had practiced any physical activity (except running) for more than a year with a frequency training of at least three times a week. Participants with concomitant musculoskeletal injuries, with underlying neurological/neuromuscular disorders, who had used anabolic steroids or steroidal anti-inflammatories or antibiotics from the fluoroquinolone group in the four weeks before the evaluation, and who had delayed muscle pain on the day of the assessment were excluded from both groups [20]. For women in both groups who have a menstrual cycle, whether regular, irregular, or under contraceptive use, data collection was not conducted 5 days before, during, or 5 days after the menstrual period [21–25]. Participants were recruited from September 2023 to May 2024 through competitive triathlon events.

### 2.3. Data Collection

Data collection began with a questionnaire to obtain identification and general data such as age (years), body mass (kg), height (m), body mass index (kg/m^2^), history of injury, medications/supplements, and training information.

Subsequently, the stiffness of the Achilles tendon, soleus, medial gastrocnemius, and lateral gastrocnemius was assessed using SWE. All evaluations were performed by a single trained and qualified evaluator to ensure consistency and minimize interoperator variability. Data collection was performed using an ultrasound (US) system (ACUSON Redwood Ultrasound System—Siemens Healthineers, Erlangen, Germany) with a linear transducer (10-L4 MHz). Elastography settings included a scale ranging from 0 to 300 kPa, with gain optimized for musculoskeletal evaluations. For the Achilles tendon, the settings were configured with a high frequency, smoothing level set to 3, gain at −3 dB, and persistence at level 3. For muscle evaluations, the preset was used with a high frequency, smoothing level set to 3, gain at 9 dB, and persistence also at level 3. These standardized settings ensured consistent image acquisition and reliable stiffness measurements across all evaluations.

**Figure 1:**
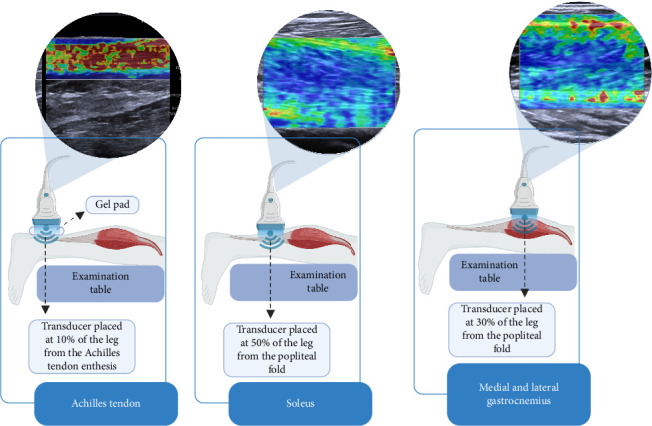
Transducer positioning for the elastography evaluation of Achilles tendon and calf muscles. (a) Evaluation of the Achilles tendon, using a PAD for better coupling with the skin; (b) evaluation of the soleus muscle; (c) evaluation of the medial and lateral gastrocnemius. The center of the muscles previously visualized in a transverse image at this region.

Participants were positioned prone on an examination table, with their knees extended and ankles in a relaxed position, allowing the feet to hang freely off the table without support or voluntary muscle contraction (Figures 1(a), 1(b), and 1(c)). This setup ensured a neutral angle of the ankle joint, avoiding active dorsiflexion or plantarflexion. To control potential confounding variables, participants were instructed to refrain from intense physical activity for at least 8 h before the evaluation. Additionally, all assessments were conducted in the morning to ensure consistent testing conditions and reflect baseline mechanical properties. Three US images were obtained for the Achilles tendon and each of the calf muscles.

**Figure 2:**
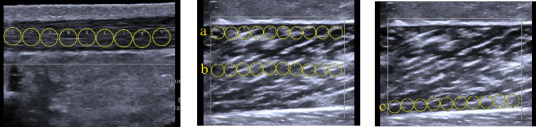
Elastography image of the Achilles tendon and medial gastrocnemius muscle, demonstrating the distribution of regions of interest (ROIs) during the examination: (a) image of the Achilles tendon demonstrating the distribution of 9 ROIs of 3 mm along the tendon; (b) image of the medial gastrocnemius muscle demonstrating the distribution of 20 ROIs of 3 mm, with 10 bordering the superficial aponeurosis (“A,” superficial layer) and 10 in the central region of the muscle (“B,” middle layer); (c) image of the medial gastrocnemius muscle, demonstrating the distribution of 10 ROIs bordering the deep aponeurosis (“C,” deep layer).

Achilles tendon measurements: The US probe was positioned perpendicular to the skin and longitudinally over the Achilles tendon, at 10% of the leg length near its insertion at the calcaneal tuberosity (Figure 1(a)). A gel pad (Hill Laboratories) was placed between the skin and the transducer to ensure acoustic coupling without exerting excessive pressure on the tissue. For each image, nine circular regions of interest (ROIs) measuring 3 mm in diameter were manually positioned along the tendon (Figure 2(a)). The total stiffness (kPa) of the Achilles tendon was calculated as the mean of the nine ROIs across three images [26, 27].

Calf muscle measurements: The US probe was positioned perpendicular to the skin and longitudinally to the muscle fibers. For the soleus, measurements were taken at 50% of the distance between the popliteal crease and the lateral malleolus (Figure 1(b)). For the medial and lateral gastrocnemius, measurements were taken at 30% of this distance (Figure 1(c)). The probe was covered with a water-soluble transmission gel to ensure consistent contact. Thirty ROIs of 3 mm in diameter were distributed across the superficial, middle, and deep muscle layers. Specifically:

• 10 ROIs were placed adjacent to the superficial aponeurosis (superficial layer) without contact (Figure 2(b)).
• 10 ROIs were positioned in the central region of the muscle (middle layer) (Figure 2(b)).
• 10 ROIs were placed adjacent to the deep aponeurosis (deep layer) without contact (Figure 2(c)).

Due to the US system's limitation of displaying 20 ROIs at a time, muscle evaluations were performed in two stages. The stiffness (kPa) of each layer was calculated as the mean of the 10 ROIs across three images. Total muscle stiffness was determined by averaging the 30 ROIs from the three images obtained for each muscle.

This standardized distribution of ROIs aimed to minimize variability caused by measurement depth and provided a comprehensive assessment of stiffness across the hierarchical structure of the muscles. Additionally, it reduced potential interference from surrounding tissues, such as myofascia, ensuring consistent and reliable conditions for comparative analysis between layers and participants.

### 2.4. Sample Size

A posteriori sample size calculation was conducted using the eta-partial square value (*η*^2^) of the group-by-layer interaction for soleus stiffness (*η*^2^ = 0.22) obtained after data collection of 42 subjects. In G⁣^∗^Power software, we used repeated-measures ANOVA, within-between interaction, with the following parameters: effect size *f* = 0.53 (obtained using *η*^2^ = 0.22); level of significance = 0.05; power = 99%; number of groups = 4 (MT x FT x MC x FC); and number of measurements = 3 (superficial × middle × deep). The G⁣^∗^Power software used the effect size index (*f*) for this analysis. The effect size *f* was directly calculated from the *η*^2^ through the following formula: *f* = *pη*^2^/(1 − *η*^2^). The analysis indicated that a total sample size of 24 participants would be sufficient for the study.

### 2.5. Statistical Analysis

Data were analyzed using SPSS (Statistical Package for Social Sciences) Version 26.0. Descriptive statistics consisted of means and standard deviation for continuous variables and frequencies and percentages for categorical variables. Data normality was tested using the Shapiro–Wilk test, while homogeneity of variances was tested using Levene's test. Participant's characteristics were compared using one-way ANOVA. Between-group differences regarding total stiffness for the Achilles tendon, soleus, medial gastrocnemius, and lateral gastrocnemius were calculated using general linear models (parametric data) and generalized linear models (nonparametric data). Differences in stiffness according to layers were calculated using generalized estimation equations. Group (male triathletes x male controls x female triathletes x female controls) was used as independent factor, layer (superficial, middle, and deep) as the repeated factor, and the stiffness of the soleus, medial gastrocnemius, and lateral gastrocnemius muscles as dependent variables. General data that were different between groups were entered as covariates in the model. Bonferroni's post hoc test was used to make pairwise comparisons. A statistical significance level of *p* < 0.05 was used.

## 3. Results

### 3.1. General Data

Participants presented a mean age of 39.23 (7.00) years, body mass of 70.60 (13.14) kg, height of 169.23 (8.86) cm, and body mass index of 24.53 (3.44) kg/m^2^. Between-group differences were found for body mass (*p* < 0.001), height (*p* < 0.001), and body mass index (*p*=0.006). As expected, men were taller and had a higher body mass than women. Specifically, in relation to body mass and height, differences were found between male triathletes and female triathletes (mean difference (MD) = 19.49, 95% CI 7.74–31.21, *p* < 0.001; MD = 14.89, 95% CI 6.98–2.77, *p* < 0.001), male triathletes and female controls (MD = 11.78, 95% CI 0.04–23.51, *p*=0.049; MD = 13.53, 95% CI 5.62–21.41, *p* < 0.001), male controls and female triathletes (MD = 22.95, 95% CI 11.21–34.68, *p* < 0.001; MD = 10.59, 95% CI 2.68–18.47, *p*=0.004), and male controls and female controls (MD = 15.25, 95% CI 3.51–26.98, *p*=0.005; MD = 9.22, 95% CI 1.32–17.11, *p*=0.015). Differences in body mass index were found only between male controls and female triathletes (MD = 5.04, 95% CI 1.33–8.74, *p*=0.003) (Table 1).

### 3.2. Between-Group Differences in the Overall Stiffness of the Achilles Tendon and Calf Muscles

**Figure 3:**
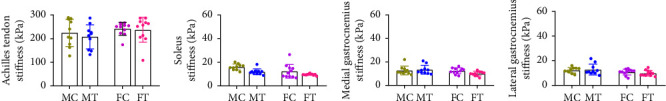
Between-group differences in the overall stiffness of the Achilles tendon and calf muscles. (a) Mean (SD) for stiffness (kPa) of the Achilles tendon; (b) mean (SD) for stiffness (kPa) of the soleus muscles; (c) mean (SD) for stiffness (kPa) of the medial gastrocnemius muscles; (d) mean (SD) for stiffness (kPa) of the lateral gastrocnemius muscles; MC, male controls; MT, male triathletes; FC, female controls; FT, female triathletes. No statistically significant differences were found (*p* > 0.05).

No between-group differences were found for the overall stiffness of the Achilles tendon and for the soleus, medial, and lateral gastrocnemius muscles measured through elastography (all *p* > 0.05) (Table 2) (Figures 3(a), 3(b), 3(c), and 3(d)).

### 3.3. Between-Group Differences in the Superficial, Middle, and Deep Layer Stiffness of the Calf Muscles

**Figure 4:**
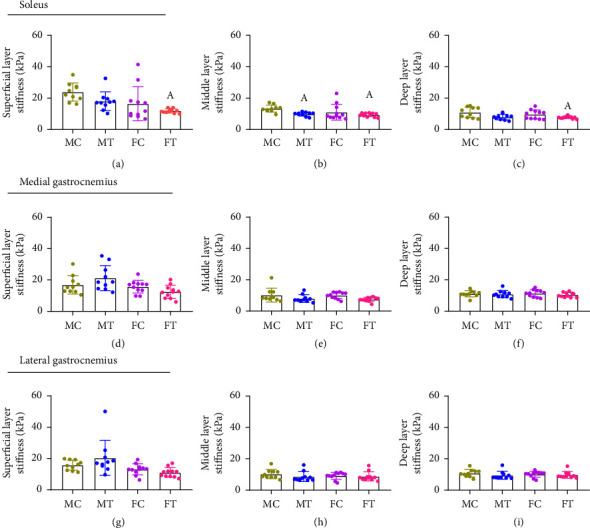
Between-group differences in the superficial, middle, and deep layer stiffness (kPa) of the soleus, medial, and lateral gastrocnemius muscles. (a) Stiffness in the superficial layer of the soleus muscle; (b) stiffness in the middle layer of the soleus muscle; (c) stiffness in the deep layer of the soleus muscle; (d) stiffness in the superficial layer of the medial gastrocnemius muscle; (e) stiffness in the middle layer of the medial gastrocnemius muscle; (f) stiffness in the deep layer of the medial gastrocnemius muscle; (g) stiffness in the superficial layer of the lateral gastrocnemius muscle; (h) stiffness in the middle layer of the lateral gastrocnemius muscle; (i) stiffness in the deep layer of the lateral gastrocnemius muscle. MC, male controls; MT, male triathletes; FC, female controls; FT, female triathletes; (A) significant difference of male controls (*p* < 0.05).

A significant group-by-layer interaction was found (*p* < 0.001) (Table 2). In the soleus muscle, the stiffness of the superficial layer was greater in the male control group compared to female triathletes (MD = 6.13, 95% CI 1.18–11.08, *p*=0.002). Also, the middle layer was greater in the male control group compared to the male triathletes (MD = 3,07, 95% CI 0.18–5.96, *p*=0.023) and female triathletes (MD = 3,05, 95% CI 0.13–5.96, *p*=0.028). Finally, the deep layer was greater in male controls compared to female triathletes (MD = 3.60, 95% CI 0.45–6.74, *p*=0.008) (Figure 4).

### 3.4. Within-Group Differences in the Superficial, Middle, and Deep Layer Stiffness of the Calf Muscles

**Figure 5:**
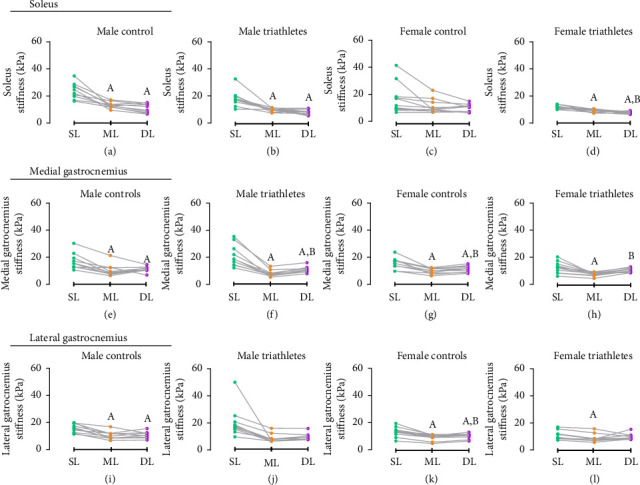
Within-group differences in the superficial, middle, and deep layer stiffness (kPa) of the soleus, medial gastrocnemius, and lateral gastrocnemius muscles. (a) Soleus of male controls; (b) soleus of male triathletes; (c) soleus of female controls; (d) soleus of female triathletes; (e) medial gastrocnemius of male controls; (f) medial gastrocnemius of male triathletes; (g) medial gastrocnemius of female controls; (h) medial gastrocnemius of female triathletes; (i) lateral gastrocnemius of male controls; (j) lateral gastrocnemius of male triathletes; (k) lateral gastrocnemius of female controls; (l) lateral gastrocnemius of female triathletes. SL, superficial layer; ML, middle layer; DL, deep layer. (A) Significant difference of superficial layer (*p* < 0.05). (B) Significant difference of middle layers (*p* < 0.05).

A significant group-by-layer interaction was found for all calf muscles (*p* < 0.001) (Table 2). In the soleus muscle, for male triathletes, stiffness was greater in the superficial layer compared to the middle (MD = 6.25, 95% CI 2.45–10.05, *p* < 0.001) and deep layers (MD = 7.68, 95% CI 3.64–11.72, *p* < 0.001) (Figure 5(b)). For male controls, stiffness was greater in the superficial layer compared to the middle (MD = 6.54, 95% CI 2.35–10.73, *p* < 0.001) and deep layers (MD = 8.03, 95% CI 2.63–13.42, *p* < 0.001) (Figure 5(a)). For female triathletes, the superficial layer was stiffer than the middle (MD = 3.46, 95% CI 0.71–6.21, *p*=0.001) and deep layers (MD = 5.49, 95% CI 3.41–7.58, *p* < 0.001), with the middle layer also greater than the deep layer (MD = 2.03, 95% CI 0.18–3.87, *p*=0.014) (Figure 5(d)).

In the medial gastrocnemius muscle, for all groups, the stiffness in the superficial layer was greater than the middle layer (MT: MD = 10.11, 95% CI 3.14–17.08, *p* < 0.001, MC: MD = 4.52, 95% CI 1.84–7.19, *p* < 0.001, FT: MD = 6.05, 95% CI 1.83–10.27, *p* < 0.001, FC: 5.64, 95% CI 1.75–9.53, *p* < 0.001) (Figures 5(e), 5(f), 5(g), and 5(h)). Additionally, for male triathletes, male controls, and female controls, the stiffness in the superficial layer was greater than in the deep layer (MT: MD = 7.78, 95% CI 0.90–14.66, *p*=0.009, MC: MD = 4.06, 95% CI 0.96–7.15, *p*=0.001, FC: MD = 4.18, 95% CI 0.42–7.95, *p*=0.012) (Figures 5(e), 5(f), and 5(g)). The deep layer was also greater than the middle layer for male triathletes, female triathletes, and female controls (MT: MD = 2.32, 95% CI 1.35–3.29, *p* < 0.001, FT: MD = 3.24, 95% CI 0.64–5.83, *p*=0.002, FC: MD = 1.45, 95% CI 0.45–2.45, *p* < 0.001) (Figures 5(f), 5(g), and 5(h)).

For the lateral gastrocnemius muscle, the superficial layer was stiffer than the middle layer in male controls (MD = 3.83, 95% CI 1.88–5.79, *p* < 0.001), female triathletes (MD = 3.18, 95% CI 1.80–4.56, *p* < 0.001), and female controls (MD = 3.88, 95% CI 1.49–6.26, *p* < 0.001) (Figures 5(i), 5(j), 5(k), and 5(l)). The superficial layer was also stiffer than the deep layer for male controls (MD = 3.43, 95% CI 1.35–5.51, *p* < 0.001) and female controls (MD = 2.81, 95% CI 0.10–5.52, *p*=0.030) (Figures 5(i) and 5(k)). Differences between the middle and deep layers were found only for female controls, with the deep layer showing greater stiffness compared to the middle layer (MD = 1.06, 95% CI 0.09–2.03, *p*=0.014) (Figure 5(k)).

## 4. Discussion

Our primary hypothesis was that triathletes would exhibit lower stiffness in the Achilles tendon and calf muscles compared to active controls. However, our results showed no significant differences in the overall stiffness between the two groups. Additionally, we hypothesized that triathletes would have lower stiffness in the superficial, middle, and deep layers of the calf muscles compared to the controls. Only minimal differences were observed between male controls and female triathletes in the soleus muscle.

Some evidence supports differences on the stiffness of the Achilles tendon and calf muscles between athletic and nonathletic populations. Collegiate endurance/team athletes and semiprofessional runners presented stiffer Achilles tendon compared to nonathletes [28, 29]. Our results align with previous studies that compared participants across various sports modalities. Specifically, no significant differences were observed when comparing track and field athletes to football, basketball, gymnastics, and ice hockey players [30], or when athletes involved in running activities were compared to nonrunners [31]. These results suggest that engaging in high-intensity physical activities like triathlon may not substantially alter the stiffness of the Achilles tendon and calf muscles.

Also, these studies did not find differences on the Achilles tendon stiffness between males and female athletes regardless of their involvement in running dominant sports [30, 31]. These results corroborate with previous research involving healthy volunteers, which also found no influence of the sex on the stiffness of the Achilles tendon and medial gastrocnemius muscle [32, 33]. It has been suggested that hormonal fluctuations, particularly those associated with the menstrual cycle, may affect stiffness [34, 35]. However, our study accounted for these factors conducting assessments outside the perimenstrual period. This methodological approach, combined with our findings, strengthens the existing evidence indicating no significant sex-based differences in tendon and muscle stiffness.

We expected that the superficial layer of the calf muscles would exhibit greater stiffness than the other layers. This hypothesis was generally confirmed across all muscles and groups studied. These results may be attributed to the distinct morphology of the muscle layers, with longer and thinner fibers present in the superficial layer compared to the shorter and thicker fibers found in the deep layer [36]. Additionally, the composition and organization of the muscle membranes, such as the epimysium and perimysium, likely contribute to these mechanical differences. The epimysium, rich in type I collagen, contrasts with the more flexible type III collagen predominant in the perimysium and endomysium [37]. Therefore, our results corroborate with the heterogeneous arrangement of muscle fibers reported by Gillies and Lieber, [19], which results in varying mechanical properties across muscle layers.

The findings of this study carry significant scientific and clinical implications. They suggest that the high-intensity endurance training typical of triathlon athletes does not necessarily induce positive or detrimental changes in tendon or muscle stiffness when compared to individuals engaged in general physical activity. This is particularly noteworthy given that previous research has indicated that pathological tendons exhibit reduced stiffness compared to healthy tendons due to structural and degenerative changes [38–40]. It is essential to recognize that stiffness adaptations are influenced by multiple factors and cannot be attributed to the specific demands of a given sport. Additionally, our findings suggest that calf muscle stiffness varies by layer, with the superficial layer exhibiting greater stiffness than the middle and deep layers. This distinction is significant for research, as most studies tend to assess the overall stiffness of the calf muscles without considering the differences in stiffness across individual layers. Also, such differences may reflect adaptations for energy storage and mechanical support. For clinicians, preventive programs for reducing calf injuries may benefit from a multimodal approach. This strategy should include interventions targeting the increased stiffness of superficial layers (e.g., soft tissue techniques) while addressing the reduced stiffness of middle and deep layers (e.g., eccentric exercises).

Some limitations should be outlined. The US used in this study has a limited stiffness measurement range (0–300 kPa). For some participants, we reached this upper limit in several ROIs, suggesting that the actual stiffness may have exceeded the maximum measurable value. Additionally, our participants were diverse in terms of training characteristics, which may have introduced variability in our results; similar challenges have been noted by Siu et al. [41]. Another limitation is the operator dependency of this method, which may have introduced potential variability in the results. Finally, the cross-sectional design prevents the establishment of causal relationships between triathlon practice and tendon/muscle stiffness.

Future research on triathletes should assess tendon and muscle stiffness under various conditions, such as after intense training and competition [41, 42]. Evaluating stiffness in different ankle positions (relaxed, dorsiflexed at 90°, and during maximal voluntary contraction) is also crucial, as joint position and loading influence stiffness [30, 43]. Elastography could serve as a monitoring tool throughout training and competition, enabling personalized training adjustments and early interventions to optimize performance, injury prevention, and return to sport.

## 5. Conclusion

Our results indicate that triathlon practice may not lead to significant differences in overall tissue stiffness compared to other types of physical activity. However, a detailed analysis of the muscle layers revealed important differences between the superficial, middle, and deep layers of the calf muscles. These findings highlight the importance of a segmented approach in muscle assessment using elastography, providing a deeper understanding of specific adaptations to different physical stimuli, such as triathlon.

## Figures and Tables

**Table 1:** Mean (SD) values for anthropometric characteristics (*n* = 42).

	MT (*n* = 10)	MC (*n* = 10)	FT (*n* = 11)	FC (*n* = 11)	*p*
Age (y)	39.60 (5.21)	39.40 (8.97)	40.63 (6.28)	37.36 (7.67)	0.753
Body mass (kg)	77.96 (7.88)	81.43 (11.32)	58.48 (7.59)	66.18 (11.17)	< 0.001⁣^∗^
Height (cm)	177.70 (9.51)	173.40 (4.50)	162.81 (4.72)	164.18 (6.17)	< 0.001⁣^∗^
BMI (kg/m^2^)	24.75 (2.48)	27.03 (3.16)	21.99 (2.03)	24.60 (4.06)	< 0.05⁣^∗^

Abbreviations: BMI, body mass index; FC, female controls; FT, female triathletes; MC, male controls; MT, male triathletes.

⁣^∗^Significant between-group differences (*p* < 0.05).

**Table 2:** Mean (95% CI) for stiffness (kPa) of the calf muscles and Achilles tendon (*n* = 42).

	MT (*n* = 10)	MC (*n* = 10)	FT (*n* = 11)	FC (*n* = 11)	*p* ^∗^
Soleus					
Superficial layer	15.60 (13.43–18.12)^†,$^	18.97 (16.43–21.89)^a,†,$^	12.83 (11.93–13.80)^†,$^	16.57 (11.13–24.66)	< 0.001⁣^∗^
Middle layer	9.34 (8.38–10.41)	12.42 (11.14–13.84)^a,b^	9.37 (8.39–10.46)^¶^	11.01 (8.35–14.53)	
Deep layer	7.91 (6.95–9)	10.93 (9.40–12.72)^a^	7.33 (6.59–8.17)	9.54 (7.98–11.41)	
Total	9.43 (6.03–12.83)	13.66 (10.79–16.52)	8.54 (4.63–12.45)	11.04 (7.60–14.49)	0.470
Medial gastrocnemius					
Superficial layer	18.36 (13.81–24.42)^†,$^	15.20 (12.90–17.91)^†,$^	12.95 (10.77–15.57)^†^	15.57 (13.24–18.30)^†,$^	< 0.001⁣^∗^
Middle layer	8.25 (6.75–10.09)^¶^	10.68 (8.62–13.24)	6.90 (5.50–8.50)^¶^	9.92 (8.84–11.13)^¶^	
Deep layer	10.58 (9.04–12.37)	11.14 (9.88–12.57)	10.14 (8.87–11.59)	11.38 (10.10–12.82)	
Total	12.34 (9.25–15.43)	12.37 (9.76–14.97)	9.89 (1.81–6.34)	12.58 (9.45–15.71)	0.997
Lateral gastrocnemius					
Superficial layer	18.63 (12.57–27.60)	13.89 (12.04–16.01)^†,$^	11.65 (9.37–14.48)^†^	13.04 (11.21–15.18)^†,$^	< 0.001⁣^∗^
Middle layer	9.35 (7.61–11.48)	10.05 (8.41–12.01)	8.47 (6.39–11.22)	9.16 (8.06–10.41)^¶^	
Deep layer	10.06 (8.45–11.97)	10.45 (8.85–12.34)	9.81 (7.97–12.07)	10.22 (9.25–11.30)	
Total	11.74 (8.96–14.52)	10.94 (8.60–13.29)	9.79 (6.60–12.99)	10.68 (7.86–13.49)	0.551
Achilles tendon					
Total	215.44 (174.10–256.78)	205.16 (170.32–240.01)	246.64 (199.11–294.16)	297.56 (255.70–339.42)	0.196

*Note:* Mean (95% CI) considering body mass, height, and body mass index as covariates. Level of significance adopted *p* < 0.05.

Abbreviations: FC, female controls; FT, female triathletes; MC, male controls; MT, male triathletes.

⁣^∗^Significant group × layer interaction.

^a^Significant differences between male controls and female triathletes.

^b^Significant differences between male controls and male triathletes.

^†^Significant differences between superficial and middle layers.

^$^Significant differences between superficial and deep layers.

^¶^Significant differences between middle and deep layers.

## Data Availability

The data supporting the findings of this study are available upon request from the corresponding author.

## Nomenclature

ANOVA: Analysis of variance
BMI: Body mass index
ECM: Extracellular matrix
ESWT: Extracorporeal shock wave therapy
FC: Female controls
FT: Female triathletes
ITU: International Triathlon Union
kPa: Kilopascals
MC: Male controls
MT: Male triathletes
MVC: Maximal voluntary contraction
ROI: Region of interest
SWE: Shear wave elastography
US: Ultrasound
